# Transcriptome analysis of fungicide-responsive gene expression profiles in two *Penicillium italicum* strains with different response to the sterol demethylation inhibitor (DMI) fungicide prochloraz

**DOI:** 10.1186/s12864-020-6564-6

**Published:** 2020-02-12

**Authors:** Tingfu Zhang, Qianwen Cao, Na Li, Deli Liu, Yongze Yuan

**Affiliations:** 1 Hubei Key Laboratory of Genetic Regulation and Integrative Biology, School of Life Sciences, Central China Normal University, Wuhan, 430079 China; 2 Yunnan Higher Education Institutions, College of Life Science and Technology, Honghe University, Mengzi, 661199 China

**Keywords:** Transcriptome, *Penicillium italicum*, Demethylation inhibitor (DMI)-resistance, Prochloraz-responsive genes

## Abstract

**Background:**

*Penicillium italicum* (blue mold) is one of citrus pathogens causing undesirable citrus fruit decay even at strictly-controlled low temperatures (< 10 °C) during shipping and storage. *P. italicum* isolates with considerably high resistance to sterol demethylation inhibitor (DMI) fungicides have emerged; however, mechanism(s) underlying such DMI-resistance remains unclear. In contrast to available elucidation on anti-DMI mechanism for *P. digitatum* (green mold), how *P. italicum* DMI-resistance develops has not yet been clarified.

**Results:**

The present study prepared RNA-sequencing (RNA-seq) libraries for two *P. italicum* strains (highly resistant (Pi-R) versus highly sensitive (Pi-S) to DMI fungicides), with and without prochloraz treatment, to identify prochloraz-responsive genes facilitating DMI-resistance. After 6 h prochloraz-treatment, comparative transcriptome profiling showed more differentially expressed genes (DEGs) in Pi-R than Pi-S. Functional enrichments identified 15 DEGs in the prochloraz-induced Pi-R transcriptome, simultaneously up-regulated in *P. italicum* resistance. These included ATP-binding cassette (ABC) transporter-encoding genes, major facilitator superfamily (MFS) transporter-encoding genes, ergosterol (ERG) anabolism component genes *ERG2*, *ERG6* and *EGR11* (*CYP51A*), mitogen-activated protein kinase (MAPK) signaling-inducer genes *Mkk1* and *Hog1*, and Ca^2+^/calmodulin-dependent kinase (CaMK) signaling-inducer genes *CaMK1* and *CaMK2*. Fragments Per Kilobase per Million mapped reads (FPKM) analysis of Pi-R transcrtiptome showed that prochloraz induced mRNA increase of additional 4 unigenes, including the other two *ERG11* isoforms *CYP51B* and *CYP51C* and the remaining kinase-encoding genes (i.e., *Bck1* and *Slt2*) required for Slt2-MAPK signaling. The expression patterns of all the 19 prochloraz-responsive genes, obtained in our RNA-seq data sets, have been validated by quantitative real-time PCR (qRT-PCR). These lines of evidence in together draw a general portrait of anti-DMI mechanisms for *P. italicum* species. Intriguingly, some strategies adopted by the present Pi-R were not observed in the previously documented prochloraz-resistant *P. digitatum* transcrtiptomes. These included simultaneous induction of all major *EGR11* isoforms (*CYP51A*/*B*/*C*), over-expression of *ERG2* and *ERG6* to modulate ergosterol anabolism, and concurrent mobilization of Slt2-MAPK and CaMK signaling processes to overcome fungicide-induced stresses.

**Conclusions:**

The present findings provided transcriptomic evidence on *P. italicum* DMI-resistance mechanisms and revealed some diversity in anti-DMI strategies between *P. italicum* and *P. digitatum* species, contributing to our knowledge on *P. italicum* DMI-resistance mechanisms.

## Background

*Penicillium digitatum* (green mold) and *P. italicum* (blue mold) are well known as the predominant citrus pathogens causing postharvest diseases during fruits storing and transportation. The resulted economic losses are so great that aroused enormous attentions all over the world [1]. The sterol demethylation inhibitor (DMI) fungicides, such as imazalil and prochloraz, have been widely applied to control citrus molds [2–6]. However, resistance to these DMI fungicides has frequently occurred for the *Penicillium* molds in the past decade, especially for *P. digitatum* isolates with high DMI-resistance [5, 7], considerably reducing the efficacy of the fungicides. Up to date, we have got some understanding on the mechanism of azole fungicide resistance in *P. digitatum* [8–13]. However, little information is available to explain *P. italicum* resistance induced by the DMI fungicides. It would be theoretically important to address molecular background of *P. italicum* isolates causing their DMI resistance.

The mechanism of fungal DMI-resistance involves strategies targeting ergosterol-biosynthesis enzymes. The site mutations in CYP51s (*ERG11*-encoding proteins) can alter drug-target interactions and increase DMI-resistance for various fungal pathogens, as reported in the model yeast *Saccharomyces cerevisiae* [14–16], the clinical pathogens *Candida albicans* [17–20] and *Aspergillus fumigatus* [21–23], and the plant pathogens *Mycosphaerella graminicola* [24, 25], *Monilinia fructicola* [26] and *P. digitatum* [27]. Fungal resistance to DMIs can also be ascribed to over-expression of CYP51s, especially by some enhancer elements [9, 27–33]. In addition to *CYP51*s, recently, other genes encoding fungal ergosterol biosynthesis-related enzymes have been proposed to be potential targets, including *ERG2* (encoding C^− 8^ sterol isomerase) [34–36] and *ERG6* (encoding C^− 24^ sterol methyltransferase) [37–40]. The importance of both *ERG2* and *ERG6* to cycloheximide resistance for *S. cerevisiae* has also been genetically emphasized [41].

Fungal DMI-resistance has also been ascribed to specific drug-transporter proteins that can reduce fungicide accumulation in fungal cells, including ATP-binding cassette (ABC) transporter family proteins, major facilitator superfamily (MFS) proteins, and multidrug and toxic compound extrusion (MATE) family proteins. ABC transporters have been functionally characterized in many fungal pathogens including green mold and verified to be up-regulated in their fungicide resistance [42–54]. MFS proteins constitute another class of broad-spectrum transporters to develop fungal DMI-resistance, including CaMDRl in *C. albicans* [55], MgMfsl in wheat pathogen *Mycosphaerella graminicola* [56], and PdMFS1 and PdMFS2 in *P. digitatum* strains [57, 58]. Unlike ABC and MFS transporters, MATE proteins function predominantly in bacterial drug-resistance [59–61]. To date, the MATE contribution to fungal drug-resistance was only reported in the ectomycorrhizal fungus *Tricholoma vaccinum* [62] and the citrus pathogenic fungus *P. digitatum* [11].

Fungicide resistance is further associated with particular protein kinase signaling and calcium (Ca^2+^) signaling. The mitogen-activated protein (MAP) kinase signaling pathways, ubiquitously found in eukaryotes (from yeasts to various pathogenic fungi), comprise a set of cascaded protein kinases, MAP kinase kinase kinase (MAPKKK), MAP kinase kinase (MAPKK) and MAP kinase (MAPK), acting in series to modulate target protein activities [63, 64]. Three major MAPK signaling pathways, Fus3/Kss1, Hog1, and Slt2, have been revealed in model yeasts [65–67] and filamentous fungi, including the citrus pathogens *Alternaria alternata* [68–71] and *P. digitatum* [72, 73], regulating pheromone/invasion processes, high osmolarity glycerol anabolism, and stress-induced cell wall remodeling, respectively. Hog1-MAPK (PdOs2)-mediated CWI signaling are involved in *P. digitatum* resistance to the fungicides iprodione and fludioxonil [72]. Hog1 homolog BcSak1 was identified in *Botrytis cinerea* and functionally required for iprodione resistance [74, 75]. FgOs2 also participated in *Fusarium graminearum* resistance to fludioxonil [76]. The latest evidence has suggested an essential role of PdSlt2 MAPK in regulating gene expression to develop azole-fungicide resistance [73]. Ca^2+^ signaling via Ca^2+^/calmodulin (CaM)-dependent kinases (CaMKs), usually linked with particular MAPK pathway(s), extensively participates in fungal responses to environmental stresses. The over-expression of CaMK2 (also named Cmk2) in the yeast *S. cerevisiae* facilitated its resistance to some azole-fungicides (e.g., dithiothreitol and miconazole) [77]. Recent studies also implied the essential role of CaMKs in protecting fungal cell wall integrity against oxidative and/or heat stresses [78–80].

RNA sequencing (RNA-seq) technology has become a powerful tool to profile transcriptomic response to reveal azole-resistance mechanism for some pathogenic fungi including prochloraz-resistant *P. digitatum* [11], voriconazole-resistant *A. fumigatus* [81], tetraconazole-resistant *Cercospora beticola* [82], tebuconazole-resistant *Fusarium culmorum* [83], and fluconazole-resistant *Candida glabrata* [84]. Our earlier report has elucidated the mechanism of *P. digitatum* resistance to DMI-fungicide prochloraz through RNA-seq analysis [11]. Nevertheless, the molecular mechanism(s) of *P. italicum* resistance to such fungicides are poorly understood. Now we have isolated two *P. italicum* strains exhibiting desirably contrasting response to common DMI fungicides including prochloraz, i.e. Pi-R (highly resistant to prochloraz with EC_50_ = 30.2 ± 1.5 mg·L^− 1^) versus Pi-S (highly sensitive to prochloraz with EC_50_ = 0.007 ± 0.002 mg·L^− 1^). The purpose of this work was to compare transcriptomic profiles between these two *P. italicum* strains with and without prochloraz treatment, to identify differentially expressed genes (DEGs) involved in the azole-class fungicide resistance, and to provide theoretical cues to explain *P. italicum* anti-azole mechanism.

## Results

### Transcriptome sequencing and assembly

In the present study, Pi-R and Pi-S were treated with or without DMI-fungicide prochloraz to prepare four RNA-seq samples, i.e., Pi-R-I, Pi-R-NI, Pi-S-I and Pi-S-NI. After Illumina sequencing, the four transcriptomic libraries contained 61,610,574, 70,012,472, 61,976,398 and 67,336,730 raw reads, respectively. By removing adaptor sequences and undesirable reads (ambiguous, low quality, and duplicated sequence reads), 58,744,798, 66,490,626, 59,134,840 and 64,262,170 clean reads were generated from the four libraries with Q30 > 90%, suggesting high quality for the present sequencing results. These clean reads were predominantly distributed in exon and intergenic regions (Additional file 4: Figure S2). Using reference genome (PHI-1) as mapping template, clean reads were assembled into 47,195,871, 54,176,219, 48,955,731 and 53,362,929 unigenes for the four libraries, respectively. All unigene expression levels in the four libraries were classified into five intervals, according to FPKM values (Table 1), and more than 50% of the total unigenes in each library were defined as highly expressed (i.e., FPKM interval ≥ 15).

**Table 1:** FPKM intervals to assess unigene expression level for four P*. italicum* RNA-seq libraries

FPKM interval	Pi-R-I	Pi-R-NI	Pi-S-I	Pi-S-NI
0~1	1930 (18.78%)	1662 (16.18%)	1746 (16.99%)	1653 (16.09%)
1~3	810 (7.88%)	725 (7.06%)	703 (6.84%)	648 (6.31%)
3~15	2296 (22.35%)	1995 (19.42%)	1874 (18.24%)	1866 (18.16%)
15~60	2831 (27.55%)	3251 (31.64%)	3314 (32.25%)	3404 (33.13%)
60~	2408 (23.44%)	2642 (25.71%)	2638 (25.67%)	2704 (26.32%)

0~1, 1~3, 3~15, 15~60, and 60~ indicate different FPKM intervals. The Table lists unigene number in each FPKM interval for each *P. italicum* RNA-seq library, and for each RNA-seq library, the percentage in bracket indicates unigene numbers in specific FPKM interval to the total unigene number

### Identification and analysis of differentially expressed genes (DEGs)

**Fig. 1:**
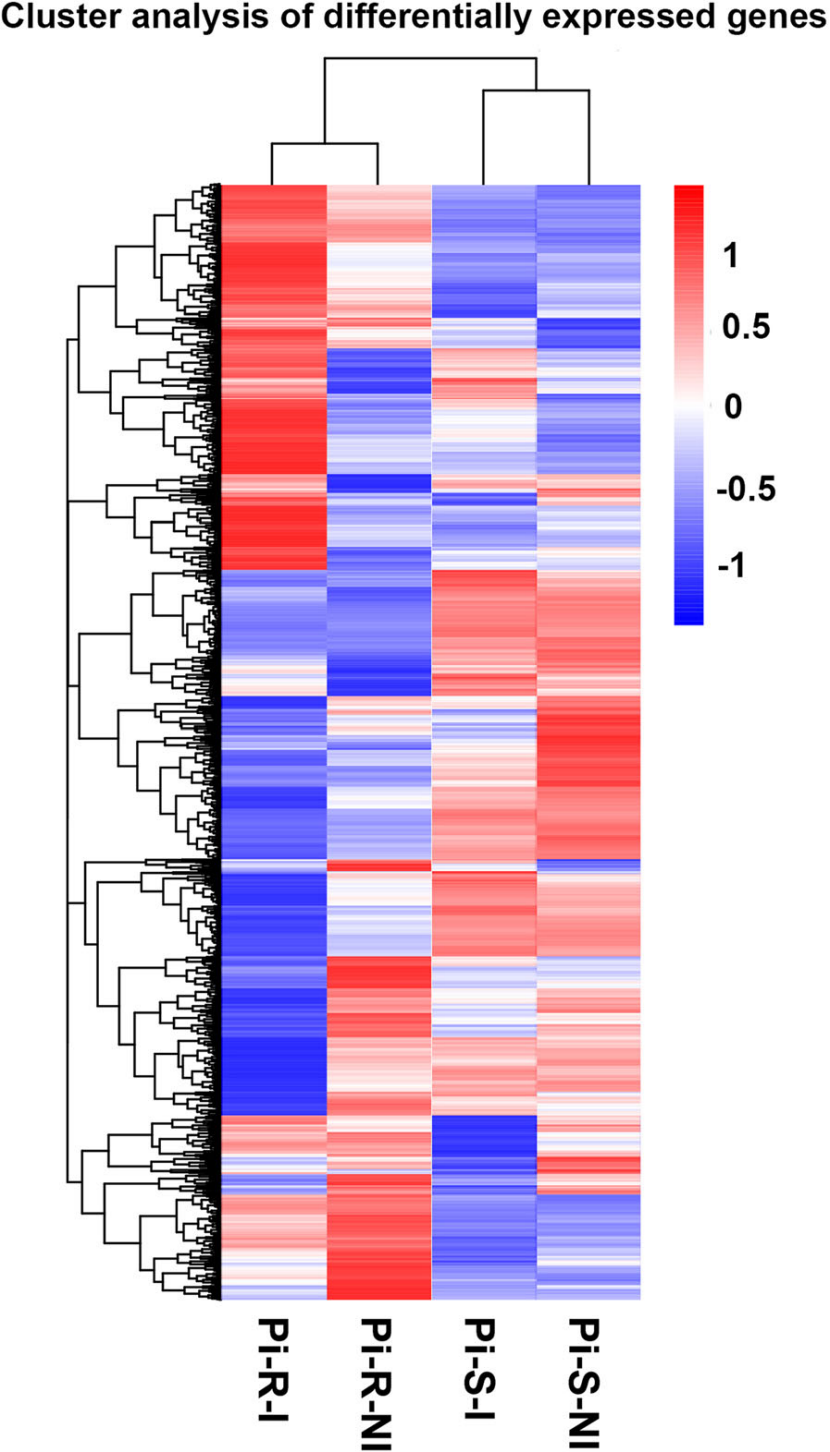
Hierarchical cluster analysis of differentially expressed genes (DEGs). Blue to red colors represent gene expression levels (i.e., FPKM values from −1 to 1)

Based on the above FPKM values, hierarchical cluster (i.e., heat map) analysis was performed to visualize DEG profiles between Pi-R-I, Pi-R-NI, Pi-S-I and Pi-S-NI libraries (Fig. 1). Pi-R and Pi-S were gathered into two independent groups each containing two clusters (i.e., with and without prochloraz induction). Noticeably, prochloraz induced more dramatic change in gene expression profile between Pi-R-I and Pi-R-NI than between Pi-S-I and Pi-S-NI, suggesting the involvement of more DEGs in Pi-R response to prochloraz.

**Fig. 2:**
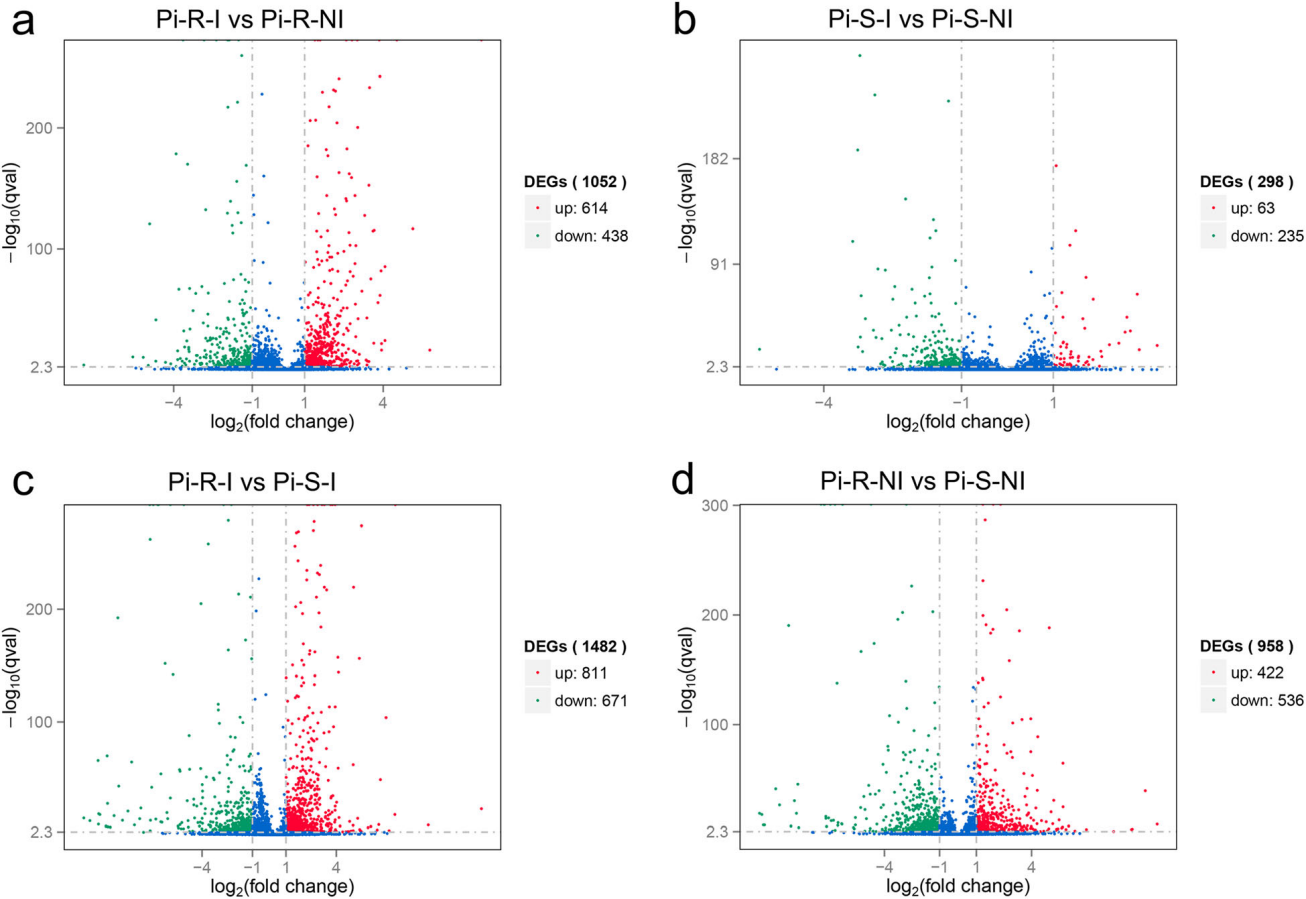
Volcano plot of DEGs in the comparison between Pi-R-I and Pi-R-NI (a), Pi-S-I and Pi-S-NI (b), Pi-R-I and Pi-S-I (c), and Pi-R-NI and Pi-S-NI (d). X-axis indicates log_2_(fold change) of DEGs between each two samples. Y-axis indicates the -log_10_(*q* value) (i.e., corrected *p* value and abbreviated as qval.) of gene expression variations, and the qval. Was applied to assess statistical significance of the change of unigene expression. The up-regulated, down-regulated, and unchanged unigenes are dotted in red, green, and blue, respectively

**Fig. 3:**
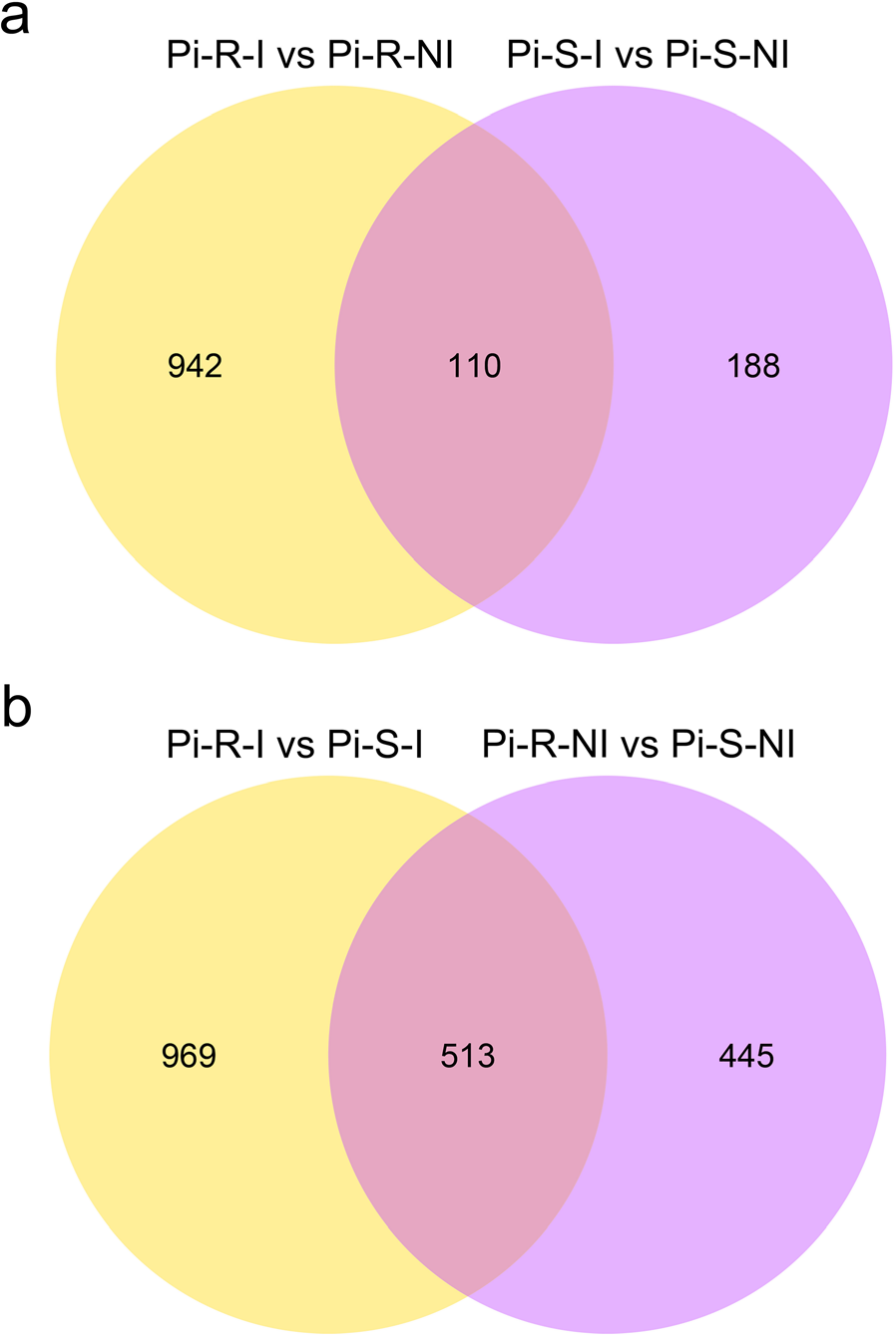
Venn diagram of DEGs shared in DEG groups Pi-R-I vs Pi-R-NI and Pi-S-I vs Pi-S-NI (a) and DEG groups Pi-R-I vs Pi-S-I and Pi-R-NI vs Pi-S-NI (b). Yellow circle stands for number of DEGs between Pi-R-I and Pi-S-I (a) and between Pi-R-I and Pi-R-NI (b). Purple circle represents number of DEGs between Pi-R-NI and Pi-S-NI (a) and between Pi-S-I and Pi-S-NI (b). The overlapping region comprises the DEGs shared in the two DEG groups Pi-R-I vs Pi-R-NI and Pi-S-I vs Pi-S-NI (a) and another two DEG groups Pi-R-I vs Pi-S-I and Pi-R-NI vs Pi-S-NI (b)

Further, the *q*-value 0.005 (i.e., corrected *p*-value 0.005) and an absolute value of log2(fold change) ≥ 1 were set as cut-off standard to identify DEGs between different libraries, including a) Pi-R-I vs Pi-R-NI, b) Pi-S-I vs Pi-S-NI, c) Pi-R-I vs Pi-S-I, and d) Pi-R-NI vs Pi-S-NI (Fig. 2). We identified 1) 1052 DEGs between Pi-R-I and Pi-R-NI (614 up-regulated and 438 down-regulated) (Fig. 2a and Additional file 5: Table S3), representing the drug-responsive genes in prochloraz-resistant strain; 2) 298 DEGs between Pi-S-I and Pi-S-NI (63 up-regulated and 235 down-regulated) (Fig. 2b and Additional file 6: Table S4), representing the drug-responsive genes in prochloraz-sensitive strain; 3) 1482 DEGs between Pi-R-I and Pi-S-I (811 up-regulated and 671 down-regulated) (Fig. 2c and Additional file 7: Table S5), representing difference in drug-induced gene expression between fungicide-resistant and -sensitive *P. italicum* strains; and 4) 958 DEGs between Pi-R-NI and Pi-S-NI (422 up-regulated and 536 down-regulated) (Fig. 2d and Additional file 8: Table S6), representing different genetic background between the two P*. italicum* strains. Among these DEGs, we identified a considerable amount of common-accepted target protein genes associated with DMI resistance, including cytochrome P450 genes and drug efflux pump genes (ABC and MFS genes rather than MATE genes) (Table 2). Based on the volcano plot analysis, we applied Venn diagrams to profile the DEG distribution between Pi-R-I vs Pi-R-NI and Pi-S-I vs Pi-S-NI (Fig. 3a) and between Pi-R-I vs Pi-S-I and Pi-R-NI vs Pi-S-NI (Fig. 3b). As shown in Fig. 3b, the overlap part of circles Pi-R-I vs Pi-S-I and Pi-R-NI vs Pi-S-NI comprised 513 DEGs that might represent DEGs irrelevant to prochloraz induction. In contrast, only 110 DEGs were distributed in the overlap part of circles Pi-R-I vs Pi-R-NI and Pi-S-I vs Pi-S-NI (Fig. 3a), indicating a proportion of DEGs potentially involved in prochloraz response in both resistant and sensitive *P. italicum* strains.

**Table 2:** Analysis of target protein genes associated with azole resistance among identified DEGs

Comparison between samples	Cytochrome P450	ABC	MFS	MATE
Pi-R-I vs Pi-R-NI	16	6	37	0
Pi-S-I vs Pi-S-NI	8	1	19	0
Pi-R-I vs Pi-S-I	19	12	68	0
Pi-R-NI vs Pi-S-NI	9	11	57	0

### GO and KEGG enrichments of prochloraz-responsive DEGs

**Fig. 4:**
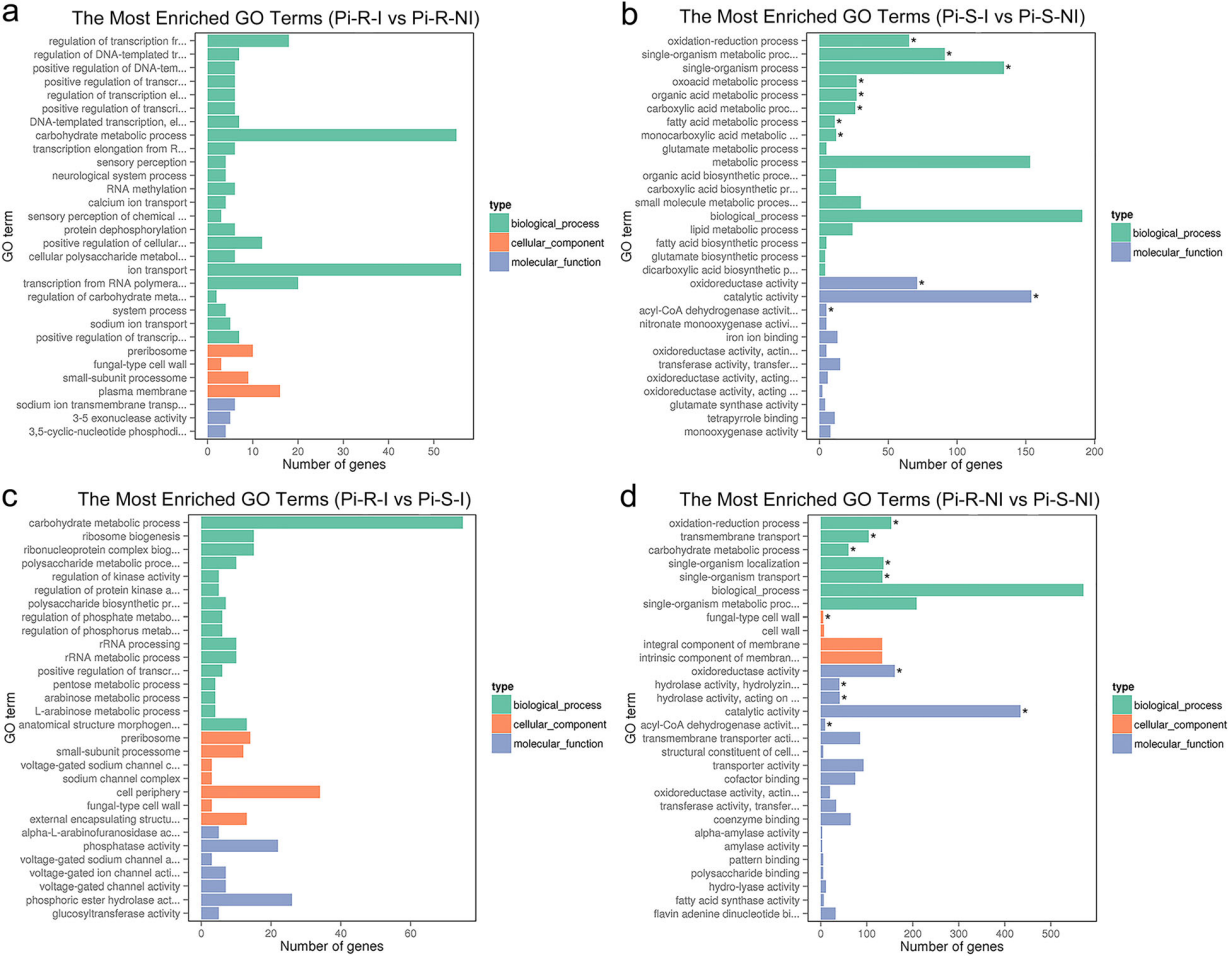
Gene ontology (GO) classifications of DEGs for Pi-R-I vs Pi-R-NI (a), Pi-S-I vs Pi-S-NI (b), Pi-R-I vs Pi-S-I (c), and Pi-R-NI vs Pi-S-NI (d). For each comparison, GO enrichment classified DEGs into three categories (types) (i.e., biological process, cellular component, and molecular function), as shown in green, orange, and purple bars, respectively. Each GO category (type) displays 30 terms (listed on Y-axis) significantly or most enriched for DEGs in the given comparisons, and X-axis indicates the number of DEGs involved in particular GO term

**Fig. 5:**
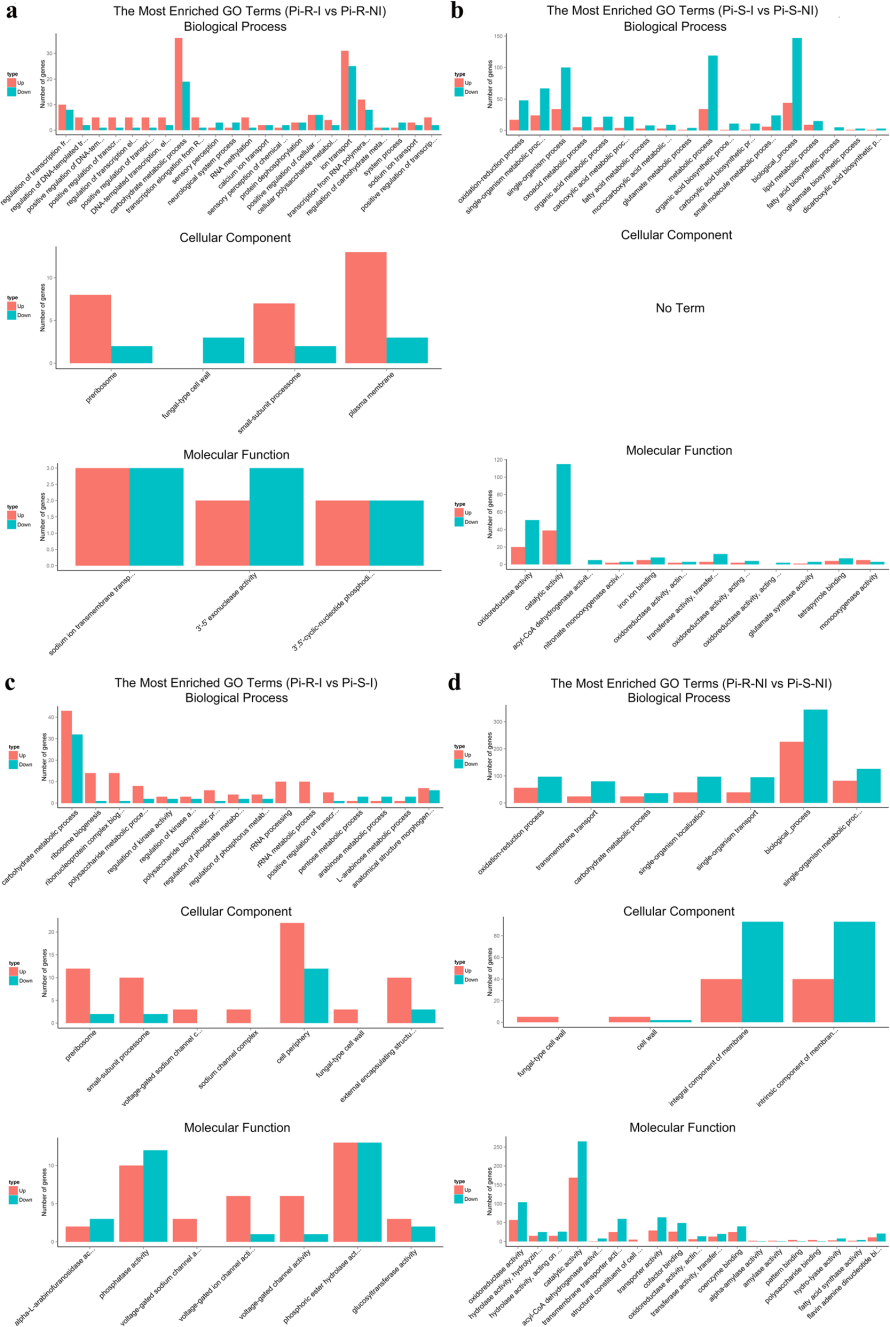
Distribution of up- and down-regulated genes in the top 30 enriched GO terms for Pi-R-I vs Pi-R-NI (a), Pi-S-I vs Pi-S-NI (b), Pi-R-I vs Pi-S-I (c), and Pi-R-NI vs Pi-S-NI (d). In each panel, from top to bottom shows three GO categories, i.e., biological process, cellular component, and molecular function, comprising the top 30 GO terms in total, and the up- and down-regulated genes in each term is represented by red and green bars, respectively. X- and Y-axis indicate GO terms and corresponding number of DEGs, respectively

The DEGs were classified into three GO categories by the Blast2GO (GOseq R package: http://www.geneontology.org), including biological process (BP), cellular component (CC), and molecular function (MF). The number of total GO terms and its distribution in the three categories for each comparison are listed in Table 3. In the comparison Pi-R-I vs Pi-R-NI (Fig. 4a), 770 DEGs were enriched into 2005 GO terms without significant enrichment. In the comparison Pi-S-I vs Pi-S-NI (Fig. 4b), 225 DEGs were enriched into 1025 GO terms with 11 significant enrichments (*q* value ≤0.05), and the top 5 terms significantly enriched were oxidoreductase activity (GO:0016491; *q* value 1.55E-07), oxidation-reduction process (GO:0055114; *q* value 3.05E-06), single-organism metabolic process (GO:0044710; *q* value 2.67E-04), catalytic activity (GO:0003824; *q* value 1.21E-03), and single-organism process (GO:0044699; *q* value 4.68E-03). In the comparison Pi-R-I vs Pi-S-I (Fig. 4c), 1086 DEGs were enriched into 2298 GO terms without significant enrichment. In the comparison Pi-R-NI vs Pi-S-NI (Fig. 4d), 711 DEGs were enriched into 1684 GO terms with 11 significant enrichments (*q* value ≤0.05), and the top 5 terms significantly enriched were oxidoreductase activity (GO:0016491; *q* value 1.73E-06), oxidation-reduction process (GO:0055114; *q* value 1.73E-06), hydrolase activity (hydrolyzing O-glycosyl compounds; GO:0004553; *q* value 1.60E-04), hydrolase activity (acting on glycosyl bonds; GO:0016798; *q* value 3.50E-04), and transmembrane transport (GO:0055085; *q* value 8.85E-04). Figure 5 reports the distribution of up- and down-regulated unigenes in the top 30 enriched GO terms for the 4 comparisons mentioned above. Interestingly, the DEGs enriched in the top 30 GO terms were found mostly up-regulated in the comparisons Pi-R-I vs Pi-R-NI and Pi-R-I vs Pi-S-I (Figs. 5a and c) and generally down-regulated in the comparisons Pi-S-I vs Pi-S-NI and Pi-R-NI vs Pi-S-NI (Figs. 5b and d).

**Table 3:** Summary of GO term distribution

Comparison between samples	GO term in total	BP	CC	MF	DEG
Pi-R-I vs Pi-R-NI	2005	1158	245	602	770
Pi-S-I vs Pi-S-NI	1025	574	105	346	225
Pi-R-I vs Pi-S-I	2298	1302	308	688	1086
Pi-R-NI vs Pi-S-NI	1684	910	214	560	711

The Table lists term numbers in GO enrichment and in the three GO categories, i.e., Biological Process (BP), Cellular Component (CC), and Molecular Function (MF), for each comparison in the present study, and correspondingly, also lists differentially expressed gene (DEG) numbers

Importantly, the up-regulated DEGs mapped to specific GO terms included a number of typical genes related to fungicide resistance. As summarized in Table 4, drug-pump genes (*ABC1*, *ABC2*, *MFS1*, *MFS2*, *MFS3* and *MFS4*, mapped to GO:0016020 (membrane)), drug-target P450 gene (*CYP51A*, mapped to GO:0055114 (oxidation-reduction process)), steroid biosynthesis-related genes (*ERG2* and *ERG6*, mapped to GO:0006694 (steroid biosynthetic process)) and MAPK/calcium signaling-related genes (*Mkk1*, *Hog1, CaMK1*, *CaMK2* and *EF-hand1*, mapped to GO:0016301 (kinase activity) and GO:0005509 (calcium ion binding)) were up-regulated in prochloraz-treated Pi-R, as compared to drug-untreated Pi-R or to drug-treated Pi-S. In contrast, most of these prochloraz-responsive DEGs, except for *CYP51A*, were down-regulated or unchanged in prochloraz-treated Pi-S, comparing to untreated Pi-S. GO enrichment also indicated lower transcript abundance of some of these prochloraz-responsive DEGs in Pi-R when compared with Pi-S under fungicide-free conditions (Table 4), including *ABC2*, *MFS1*, *MFS2*, *MFS4*, and *CaMK2*. The GO-term map distribution (i.e., hit and ranking records) of the prochloraz-responsive DEGs mentioned above was summarized in Additional file 9: Table S7.

**Table 4:** Summary of GO-enriched DEGs associated with prochloraz resistance

GO ID (term)	DEG ID	DEG name (GO-annotated)	Fold change (log2) of DEG expression in the comparison below			
			Pi-R (I/NI)	Pi-S (I/NI)	I (Pi-R/Pi-S)	NI (Pi-R/Pi-S)
GO:0016020 (membrane)	PITC_032590	*ABC1*	7.746	/	7.523	/
	PITC_006400	*ABC2*	1.393	/	/	−1.385
	PITC_098100	*MFS1*	3.880	/	1.838	−1.709
	PITC_012240	*MFS2*	3.853	/	/	−3.011
	PITC_056240	*MFS3*	2.602	/	1.922	/
	PITC_091150	*MFS4*	2.069	−1.512	1.225	−2.336
GO:0055114 (oxidation-reduction process)	PITC_083360	*CYP51A*	2.865	2.425	/	/
GO:0006694 (steroid biosynthetic process)	PITC_027000	*ERG2*	2.002	/	1.422	/
	PITC_014340	*ERG6*	1.757	/	1.365	/
GO:0016301 (kinase activity)	PITC_088710	*Mkk1*	2.603	/	2.143	/
	PITC_062470	*Hog1*	/	−1.215	1.801	/
	PITC_087700	*CaMK1*	2.402	/	/	−1.237
	PITC_025800	*CaMK2*	1.138	−1.752	2.578	/
GO:0005509 (calcium ion binding)	PITC_033750	*EF-hand1*	1.510	/	1.543	/
	PITC_036760	*EF-hand2*	−1.814	/	−1.504	/

The abbreviated names for the selected DEGs are listed as: *ABC1*, *CDR ABC transporter 1*; *ABC2*, *ABC transporter 2* (integral membrane type); *MFS1*, *major facilitator superfamily protein 1*; *MFS2*, *major facilitator superfamily protein 2*; *MFS3*, *major facilitator superfamily protein 3*; *MFS4*, *major facilitator superfamily protein 4*; *CYP51A*, *cytochrome P450 51A*; *ERG2*, *ergosterol biosynthesis methyltransferase 2*; *ERG6*, *ergosterol biosynthesis methyltransferase 6*; *Mkk1*, *MAP kinase kinase* (i.e., *Mkk1,2*); *Hog1*, *MAP kinase* (i.e., *MpkC*); *CaMK1*, *calcium/calmodulin-dependent protein kinase 1*; *CaMK2*, *calcium/calmodulin-dependent protein kinase 2*; *EF-hand1*, *calcium-binding EF-hand 1*; *EF-hand2*, *calcium-binding EF-hand 2*. Pi-R (I/NI), Pi-S (I/NI), I (Pi-R/Pi-S) and NI (Pi-R/Pi-S) indicate comparisons Pi-R-I vs Pi-R-NI, Pi-S-I vs Pi-S-NI, Pi-R-I vs Pi-S-I and Pi-R-NI vs Pi-S-NI, respectively. The log2(fold change) of DEG expression was generated from original data in DEG information lists (Table S2–5), and the slash ‘/’ indicates the unfeasibility to generate log2(fold change) when the DEG is not included in the comparison

Further, KEGG enrichment was applied to identify pathways associating the prochloraz-responsive DEGs with resistance mechanisms. In the present four comparisons, KEGG analysis enriched prochloraz-responsive DEGs into only two pathways, i.e., steroid biosynthesis (KEGG ID: pcs00100; *q* value = 0.013) and MAPK signaling pathway (KEGG ID: pcs04011; *q* value = 0.021) (Table 5): the former pathway exclusively included up-regulated DEGs, i.e., *CYP51A* (PITC_083360) in the comparisons Pi-R (I/NI) and Pi-S (I/NI), *ERG2* (PITC_020620) in the comparisons Pi-R (I/NI) and Pi-S (I/NI), and *ERG6* (PITC_014340) in the comparisons Pi-R (I/NI) and I (Pi-R/Pi-S); while the latter pathway included 1) up-regulated DEGs (i.e., *Mkk1* and *Hog1*) in Pi-R-involved comparisons, i.e., Pi-R (I/NI) and I (Pi-R/Pi-S) and 2) down-regulated DEG (i.e., *Hog1*) only in comparison Pi-S (I/NI). All the KEGG-enriched DEGs, as components of metabolic and/or signal-transduction pathway(s), were well coincident with the results of GO enrichment. In other words, the present GO-enriched DEGs, if involved in specific biological pathway(s), were exclusively KEGG-included, and certainly, pathway-irrelevant genes, e.g., drug-pump genes and drug-target genes, were KEGG-excluded, without exception.

**Table 5:** Summary of KEGG enrichments for prochloraz-responsive DEGs in the present 4 comparisons

KEGG pathway (ID)	Gene name (ID)	Comparison involved	Regulated
Steroid biosynthesis (pcs00100)	*CYP51A* (PITC_083360)	Pi-R (I/NI)	Up
		Pi-S (I/NI)	Up
Steroid biosynthesis (pcs00100)	*ERG2* (PITC_020620)	Pi-R (I/NI)	Up
		Pi-S (I/NI)	Up
Steroid biosynthesis (pcs00100)	*ERG6* (PITC_014340)	Pi-R (I/NI)	Up
		I (Pi-R/Pi-S)	Up
MAPK signaling pathway-yeast (pcs04011)	*Mkk1* (PITC_088710)	Pi-R (I/NI)	Up
		I (Pi-R/Pi-S)	Up
MAPK signaling pathway-yeast (pcs04011)	*Hog1* (PITC_062470)	Pi-S (I/NI)	Down
		I (Pi-R/Pi-S)	Up

### Real-time quantitative PCR (qRT-PCR) validation of prochloraz-responsive DEGs

**Fig. 6:**
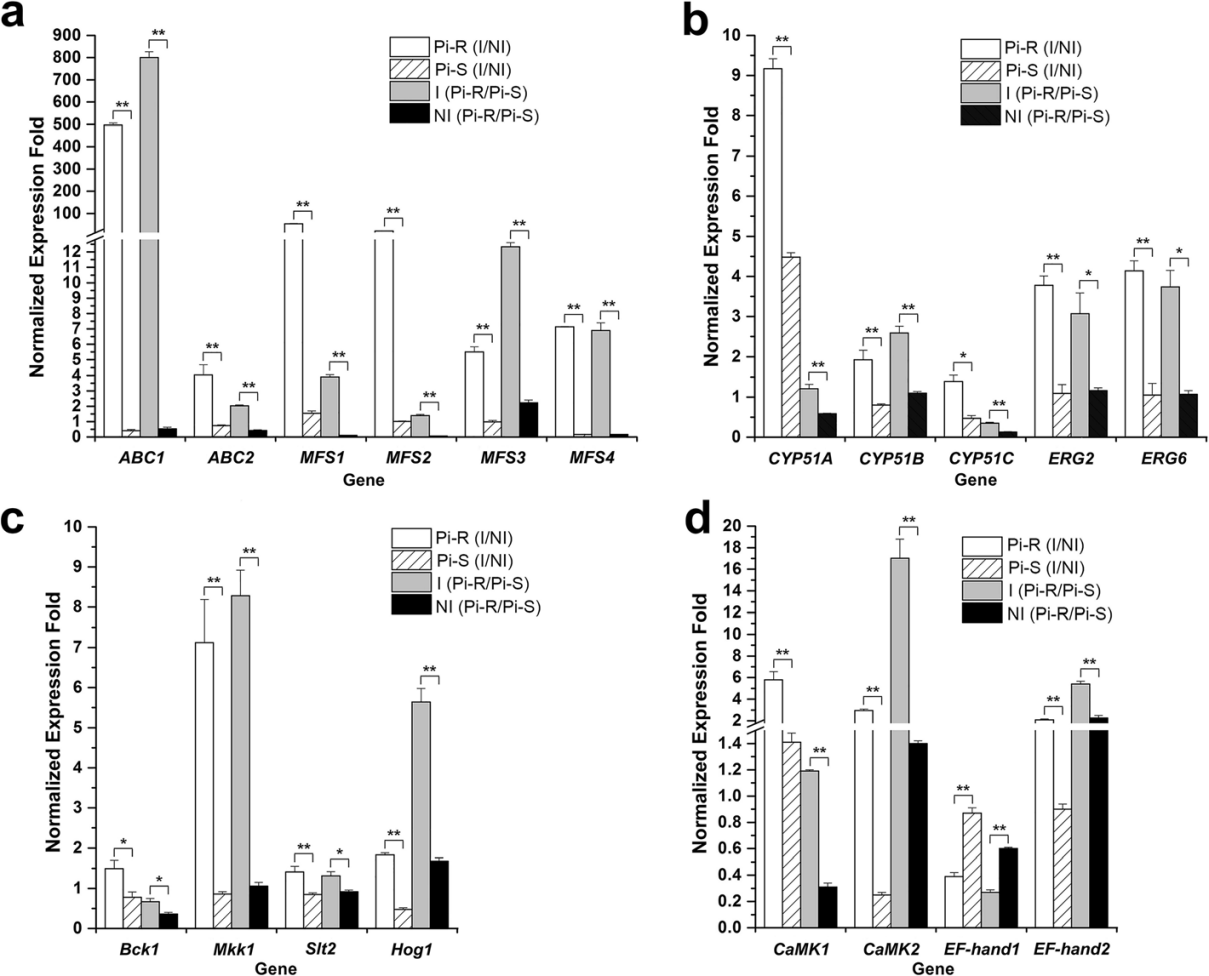
qPCR validation of 19 prochloraz-responsive DEGs including drug transporter genes (a), ergosterol biosynthesis-related genes (b), MAPK signaling pathway genes (c), and Ca^2+^ signal transduction genes (d). The housekeeping gene *β-actin* was used as internal reference to calculate the relative mRNA abundance for the selected unigenes. Relative ratios for the expression of each selected DEG were calculated as Pi-R (I/NI), Pi-S (I/NI), I (Pi-R/Pi-S), and NI (Pi-R/Pi-S). All values obtained in the qRT-PCR analysis were expressed as the mean ± SD from 5 biological repeats each containing 3 technical replicates, and independent samples *t*-test (n = 5) was applied in the SPSS Statistics 17.0 context to assess the significance of differences between the means (*p < 0.05 and **p < 0.01)

According to the GO and KEGG enrichments combined, we selected 15 prochloraz-responsive DEGs to perform qRT-PCR validation. The 15 prochloraz-responsive DEGs, never reported before, potentially involved in *P. italicum* response to DMI fungicides, included 1) drug-pump genes: *ABC1* (PITC_032590), *ABC2* (PITC_006400), *MFS1* (PITC_098100), *MFS2* (PITC_012240), *MFS3* (PITC_056240), and *MFS4* (PITC_091150); 2) ergosterol biosynthesis-related genes: *CYP51A* (PITC_083360), *ERG2* (PITC_027000), and *ERG6* (PITC_014340); 3) MAPK signaling-related genes: *Mkk1* (PITC_088710) and *Hog1* (PITC_062470); 4) Ca^2+^ signal transducer-related genes: *CaMK1* (PITC_087700), *CaMK2* (PITC_025800), *EF-hand1* (PITC_033750), and *EF-hand2* (PITC_036760). Additionally, FPKM-based unigene expression quantification combined with local Blast-based annotation revealed differential expression patterns for particular prochloraz-responsive unigenes in the present 4 comparisons, including *CYP51B* (PITC_064600), *CYP51C* (PITC_028940), *Bck1* (PITC_061930) and *Slt2* (PITC_008290) (Additional file 10: Table S8). Considering 1) functional clustering of *CYP51A*/*B*/*C* (i.e., isofroms of drug-target gene *CYP51*) and 2) cascaded association of *Bck1* (encoding MAPKKK), *Mkk1* (encoding MAPKK) and *Slt2* (encoding MAPK) in Slt2-MAPK pathway, we also performed qRT-PCR validation for the 4 prochloraz-responsive unigenes that were not included in the present DEG list for comparison (i.e., not included in Additional files 5-8: Tables S3–6). As shown in Fig. 6, the qRT-PCR expression patterns of the total 19 prochloraz-responsive DEGs (including 4 FPKM-defined DEGs) were all in agreement with the obtained RNA-seq results. Further, the qRT-PCR results using internal reference gene *β-actin* were confirmed by another dataset of qRT-PCR analysis based on a different housekeeping gene *GAPD* (Additional file 11: Figure S3).

In detail, the transcript abundance of drug-pump gene *ABC1* was strikingly increased in both Pi-R (I/NI) and I (Pi-R/Pi-S), by nearly 500- and 800-folds, respectively, while remarkably decreased in both Pi-S (I/NI) and NI (Pi-R/Pi-S); the similar (but not so strikingly) changing pattern was observed for the rest drug-pump genes including *MFS1* (Fig. 6a). When comparing Pi-R (I/NI) with Pi-S (I/NI) or comparing I (Pi-R/Pi-S) with NI (Pi-R/Pi-S), the obviously higher increasing-fold of transcript abundance was also validated for the other prochloraz-responsive genes, including typical drug-target genes (i.e., *CYP51A*/*B*/*C*) (Fig. 6b), ergosterol biosynthesis-related genes *ERG2* and *ERG6* (Fig. 6b), MAPK signaling-related genes (Fig. 6c), and Ca^2+^ signal transducer-related genes *CaMK1*, *CaMK2* and *EF-hand2* (Fig. 6d). In addition, to functionally verify particular prochloraz-responsive gene, an *mfs1*-knockout mutant (Δ*mfs1*) was constructed from its parental strain Pi-R, exhibiting obviously lower prochloraz-resistance (i.e., lower prochloraz EC_50_ value) as compared to the Pi-R wild-type (Additional file 12: Figure S4). This was a sort of preliminary observation from the present RNA-seq analysis, and further biological support is in process.

## Discussion

In the past decades, conventional synthetic DMI-fungicides, such as prochloraz and imazalil, were widely applied to control *Penicillium* decay, but undesirably, a considerable number of resistant isolates including *P. digitatum* and *P. italicum* strains have developed [5–7]. The mechanisms underlying DMI-fungicide resistance have been elucidated for *P. digitatum* species by transcriptomic analysis [11]. However, how to develop DMI-resistance in *P. italicum* species is still not clear, might due to rare opportunity to find highly DMI-resistant *P. italicum* strain(s). The EC_50_ values of *P. italicum* isolates towards DMI-fungicide(s) (e.g., imazalil), published to date, were ≤ 0.92 ± 0.09 mg·L^− 1^, no more than moderate resistance level [5, 85]. Nevertheless, some recent investigations have suggested evolutional potential to develop high DMI-resistance in *P. italicum* species [85–87]. Now we have isolated a *P. italicum* strain (Pi-R) with extremely high resistance to some common DMI-fungicides including prochloraz (Additional file 1: Figure S1 and Additional file 2: Table S1). We believed that this strain could be useful to investigate DMI-fungicide resistance mechanism in *P. italicum*.

Fungal resistance to azole-fungicides including a number of DMI-fungicides has been usually ascribed to over-expression of specific drug-efflux pumps such as ABC and MFS transporters [8, 42–50, 53, 54, 57, 58, 88]. Specially, ABC and MFS transporter-encoding genes, each containing multiple isoforms, were reported to be simultaneously up-regulated in the prochloraz-resistant *P. digitatum* [11]. The similar up-regulation of multiple ABC and MFS gene members (i.e., *ABC1*–*2* and *MFS1*–*4*) was also observed in the prochloraz-treated Pi-R rather than Pi-S (Table 4 and Fig. 6a), indicating the need of drug-efflux pumps to develop DMI-resistance in *P. italicum*. The evidence that the knockout of *mfs1* (i.e., MFS1-encoding gene) in Pi-R decreased the fungal resistance to prochloraz confirmed the involvement of particular drug-efflux pump in *P. italicum* anti-DMI mechanism (Additional file 12: Figure S4), and more biological support would be required for the other drug-efflux pumps. Interestingly, regarding these drug-efflux pump-encoding genes, over-expressed in the prochloraz-induced *P. italicum* transcriptome, their orthologous genes in the prochloraz-induced *P. digitatum* transcriptome [11], as shown in Additional file 13: Table S9, were not responsive or negatively responsive to the DMI-fungicide treatment. Such different isogene responsive profiles suggested a special drug-pump preference for different *Penicillium* species to develop DMI-fungicide resistance. Here we provide the first evidence that the two *Penicillium* species (i.e., green mold and blue mold) with close evolutionary association did differ in selecting drug-transporters to support their DMI resistance, and the mechanism(s) underlying need further research.

More interestingly, regarding another class of drug-pump-encoding genes, i.e., *MATE*s, none was found to be differentially expressed in the prochloraz-induced Pi-R and Pi-S transcriptomes (Table 2). In contrast, Liu et al. [11] reported up-regulation of three MATE transporter-encoding genes (*MATE1*–*3*) in *P. digitatum* resistance to prochloraz. The orthologous gene of *PdMATE3* has been identified in the present Pi-R and Pi-S genomes, however, no mRNA transcripts of this gene was detected in the prochloraz-treated *P. italicum* strains. This might suggest transcriptional irrelevance of MATE transporter with *P. italicum* resistance to prochloraz. Unlike ABC and MFS transporters, MATE transporters are more associated with bacterial drug-resistance [59–62]. To date, the transcriptional response of *MATE* isogenes to DMI fungicides was only documented in prochloraz-resistant *P. digitatum* transcriptomes [11]. But such transcriptomic response of MATE transporter-encoding genes did not occur in the present *P. italicum* transcriptomes. Thus a potential debate on real function of MATE family member(s) in citrus *Penicillium* pathogens’ resistance to DMI-fungicides would be an interesting study topic.

Over-expression of *ERG11*s, the P450-dependent sterol 14α-demethylase (CYP51)-encoding genes (e.g., *CYP51A*/*B*/*C*), has been accepted as a primary strategy to develop fungal DMI-resistance [9, 27, 33]. Under prochloraz induction, the increasing fold of *CYP51A* in Pi-R (~ 9-fold) was more than that in Pi-S (~ 4.5-fold) (Fig. 6b), suggesting a positive correlation between *CYP51A* abundance and anti-DMI potential in *P. italicum* strains. On the other hand, the simultaneous up-regulation of *ERG11* isoforms (*CYP51A*/*B*/*C*) was observed only in prochloraz-induced Pi-R, rather than Pi-S (Table 4 and Fig. 6b). This indicated that *CYP51B* and *CYP51C* could contribute to *P. italicum* prochloraz-resistance. However, according to the prochloraz-induced *P. digitatum* transcriptome [11], the orthologous genes of *CYP51B* and *CYP51C* in the prochloraz-resistant *P. digitatum* strain were not up-regulated (Additional file 13: Table S9). The difference in choice of *ERG11*-encoding targets suggested diverse strategies for *P. italicum* and *P. digitatum* species to develop DMI-resistance. *ERG2* and *ERG6*, the other two *ERG*s in ergosterol-biosynthesis pathway, as recommended to be potential multidrug targets [89], were up-regulated in the prochloraz-treated Pi-R (Tables 4, 5 and Fig. 6b). These *ERG*s could be contributors to the *P. italicum* DMI-resistance. Previous reports have documented azole-induced mRNA increase of *ERG2* and *ERG6* [39, 81], correlating these ergosterol-biosynthesis genes with acute response of fungal membrane rigidity caused by azole-fungicides. Such *ERG*s expression profile was observed in the DMI-resistant *P. italicum*, but not in the prochloraz-resistant *P. digitatum* strain [11] (Additional file 13: Table S9). These results suggested different strategies in *ERG*s response of the two *Penicillium* species to develop DMI-resistance.

Fungal multidrug resistance has been linked with MAPK signaling pathways including Slt2-MAPK [73, 90–92]. The Slt2-MAPK middle-stream components, two MAPKK-encoding genes *ScMkk1* and *ScMkk2*, have been characterized in the model yeast *S. cerevisiae* to function in CWI signaling-mediated fungicide resistance [90, 91]. In the present study, *Mkk1* (PITC_088710), the homolog of *ScMkk1* in *P. italicum* species, was GO- and KEGG-enriched as up-regulated DEG in the prochloraz-treated Pi-R (Tables 4 and 5), indicating the involvement of Slt2-MAPK in the *P. italicum* DMI-resistance. *Bck1* (PITC_061930) and *Slt2* (PITC_008290), the MAPKKK- and MAPK-encoding genes located upstream and downstream of *Mkk1*, respectively, were both up-regulated in prochloraz-treated Pi-R (Fig. 6c and Additional file 10: Table S8). These results supported Slt2-MAPK contribution to the *P. italicum* prochloraz-resistance. Disruption of *slt2* led to increased sensitivity to DMI-fungicide imazalil in wheat pathogen *M. graminicola* [93], also suggesting Slt2-MAPK function in fungal DMI-resistance. However, *slt2*-deleted *P. digitatum* exhibited no obvious change in DMI-resistance [73], indicating that Slt2-MAPK might be irrelevant to the fungal anti-DMI strategy. Actually, according to prochloraz-resistant *P. digitatum* transcriptomes [11], the orthologs of PiSlt2-MAPK genes were not included in up-regulated DEGs (Additional file 13: Table S9). These contrasting results indicated an interesting difference in MAPK choice for different fungal pathogens to resist DMI fungicide(s). Fungal resistance to azole fungicides additionally required some cellular Ca^2+^-signaling processes via CaMKs, specially, via *CaMK1*, *CaMK2* and their homologues [78–80, 94]. Here we also identified two *CaMK* homologues *CaMK1* (PITC_087700) and *CaMK2* (PITC_025800), both up-regulated only in prochloraz-treated Pi-R (Table 4 and Fig. 6d), thus suggesting the contribution of CaMKs to fungal DMI-resistance. CaMKs mediated fungal responses to cell-wall and oxidative stresses induced by fungicides [77–80]. Thus the involvement of *CaMK*s in *P. italicum* DMI-resistance would be a strategy to cope with some azole-induced stresses. Unlike Pi-R, the prochloraz-resistant *P. digitatum* strain showed no up-regulation of *CaMK* homologues (Additional file 13: Table S9). The difference in *CaMK* response to DMI-fungicides in the two *Penicillium* species needs further research.

## Conclusions

In conclusion, the present work for the first time provided transcriptomic analysis of prochloraz-responsive gene expression profiles for two *P. italicum* strains with contrasting response to prochloraz, revealing potential mechanisms underlying *P. italicum* resistance against DMI fungicides. The strategies that *P. italicum* species adopt to overcome the azole stresses, based on DEG enrichment analysis, are summarized as 1) up-regulation of specific isogenes encoding ABC and MFS transporters, 2) simultaneous induction of all major *EGR11* isoforms including *CYP51A*/*B*/*C*, 3) over-expression of *ERG2* and *ERG6* to modulate ergosterol anabolism, and 4) concurrent mobilization of Slt2-MAPK and CaMK signaling processes to adapt azole-induced stresses. Some differences in the choice of anti-DMI strategy between *P. italicum* and *P. digitatum* species were also discussed.

## Methods

### Strains, cultivation and treatments

*P. italicum* strains Pi-R and Pi-S, used in this study, were isolated from rotten citrus fruits in local packinghouses (Yunnan Province) and orchards (Hainan Province), respectively. Pi-R exhibits dramatically high prochloraz-resistance (EC_50_ = 30.2 ± 1.5 mg·L^− 1^), while Pi-S is prochloraz-sensitive (EC_50_ = 0.007 ± 0.002 mg·L^− 1^) (Additional file 1: Figure S1). Meanwhile, Pi-R and Pi-S did show contrasting response to the other two DMI fungicides (imazalil and triadimefon) and the two benzimidazole-class fungicides (carbendazim and benomyl), according to their EC_50_ values; however, the two fungal strains were both highly sensitive to the phenylpyrrole fungicide fludioxonil (Additional file 2: Table S1). Fungal strains were routinely cultivated on potato dextrose agar (PDA) medium for 5 days to prepare respective conidial suspension (10^7^ spores mL^− 1^) as previously described [11]. Afterwards, for each fungal strain, 200 μL of conidial suspension (approximately 2 × 10^6^ spores) was incubated with 200 mL potato dextrose broth (PDB) medium for 2 days at 28 °C and 180 rpm shaking, and the resulting mycelia were treated with or without prochloraz. In detail, prochloraz was added to the PDB medium at final concentration that was in agreement with EC_50_ value for each *P. italicum* strain (i.e., 30.2 mg·L^− 1^ for Pi-R and 0.007 mg·L^− 1^ for Pi-S). Prochloraz was pre-dissolved in 100 μL DMSO, and the same volume of DMSO was added to 200 mL PDB medium to prepare control samples. The prochloraz-induced and no-induced (control) samples were cultured at the same conditions (at 28 °C and 180 rpm shaking) for 6 h before RNA extraction. The present study collected 4 samples in total for the following RNA manipulations, i.e. prochloraz-induced and no-induced Pi-R (designated as Pi-R-I and Pi-R-NI, respectively), and prochloraz-induced and no-induced Pi-S (designated as Pi-S-I and Pi-S-NI, respectively).

### RNA extraction, RNA-seq library construction and Illumina sequencing

Total RNA was extracted for the four fungal samples with three biological replicates, according to the method described before [11]. The integrity of RNA samples was first examined by 1% (w/v) agarose gel electrophoresis analysis and then confirmed by Bioanalyzer 2100 (Agilent Technologies, CA, USA). Each RNA sample was further checked by Nano-Photometer (Implen, CA, USA) to ensure its purity, and quantified using Qubit RNA Assay Kit (Life Technologies, CA, USA). Sequencing libraries were constructed according to the protocol of NEBNext Ultra™ RNA Library Prep Kit for Illumina sequencing (NEB, USA), using the amount of 3 μg RNA for each sample. Briefly, Poly (A) mRNA was purified and enriched from total RNA by oligo-dT paramagnetic beads, and fragmented to provide templates for the first- and second-strand cDNA synthesis. The cDNA products were end-repaired to be blunt fragments and adenylated at their 3′ ends. NEBNext adaptors (sequencing adapters) were ligated with the 3′-adenylated DNA fragments. Then 3 μL USER Enzyme (NEB, USA) was applied to produce double-stranded cDNA templates for polymerase chain reaction (PCR) amplification with Pfu DNA polymerase and specific primers. The obtained index-coded samples were clustered by cBot Cluster Generation System for sequencing using Illumina HiSeq X platform, and the resulting paired-end reads (raw reads) in ~ 150 bp length were deposited at NCBI database under accession number PRJNA421419.

### Reads mapping to the reference genome

Raw reads stored in fastq format after the Illumina sequencing were first processed through in-house perl scripts to thoroughly remove low quality reads, as described before [11], to generate clean reads with high quality that were assessed by parameters Q20, Q30 and GC content. The clean reads were mapped to *P. italicum* PHI-1 reference genome (GenBank accession number: JQGA01000000) [95] using TopHat version 2.0.11 [96]. Prior to the reads mapping, the gene annotation files were downloaded from the website (http://genome.jgi.doe.gov/Pendi1 /Pendi1.home.html), and the reference genome was indexed by Bowtie version 2.0.6.

### Gene expression analysis and functional enrichments

Gene expression level (transcript and/or unigene abundance) in each sample was estimated using HTSeq v0.6.1, according to FPKM analysis [97], which processed based on uniquely mapped reads and thus eliminating the experimental bias due to sequencing discrepancies. Then, the read counts were adjusted by edgeR program package through one scaling normalized factor, as previously described [98], to prepare for differential gene expression analysis. To identify differentially expressed genes (DEGs) between samples, fold-changes of expression level for each gene, defined as the ratio of the RPKM values, were calculated by DEGSeq R package (1.12.0), and *P*-values were statistically corrected to assess the significance for the differences in transcript abundance according to Benjamini & Hochberg method [99]. In the present study, DEGs were selected as transcripts and/or unigenes differentially expressed with at least 2-fold change (i.e., the absolute value of log2 Fold change ≥1.0) and corrected *P*-value ≤0.005 between two groups of comparison. The identified DEGs were hierarchically clustered by Cluster 3.0 [100], and then subjected to heat-map analysis by Plotly (Montreal, Quebec) software and Venn diagram analysis at the website http://bioinfogp.cnb.csic.es/tools/venny/index.html. Further, DEGs were functionally enriched to Gene Ontology (GO) database (http://www. geneontology.org) using the GOseq R package based on Wallenius’ non-central hyper-geometric distribution [101], and also enriched to Kyoto Encyclopedia of Genes and Genomes (KEGG) public database (http://www.genome.jp/kegg/), using the KOBAS software to test the significance of enriched DEGs in particular metabolic and signal transduction pathways [102].

### Quantitative real-time PCR (qRT-PCR) validation

Nineteen unigenes related to azole-drug resistance in the present *P. italicum* transcriptoms were selected for qRT-PCR validation, including 6 drug transporter genes, 5 ergosterol biosynthesis-related genes, 4 MAPK signaling pathway-related genes, and 4 Ca^2+^ signal transducer-related genes. Total RNA was extracted from the same *P. italicum* samples used for RNA-seq, according to Fungi RNA Kit user guide (OMEGA, USA). First-strand cDNA synthesis was performed with PrimeScript™RT reagent Kit with gDNA Eraser (TaKaRa, Dalian, China), according to the manufacturer’s instructions, and the qRT-PCR was performed using a BIO-RAD CFX96 qPCR system with SYBR Green I fluorescent dye detection as previously described [11]. The specific primers used in this study are shown in Additional file 3: Table S2, and the two housekeeping genes, i.e., *β-actin* and *glyceraldehyde-3-phosphate dehydrogenase* (*GAPD*), were respectively applied as internal reference to calculate the relative mRNA abundance for the selected unigenes, according to the 2^–ΔΔCt^ method [103] with 5 biological repeats each containing three technical replicates. Relative ratios for the expression of each selected unigene were further calculated in the 4 comparison groups, including Pi-R (I/NI) (i.e., Pi-R-I relative to Pi-R-NI), Pi-S (I/NI) (i.e., Pi-S-I relative to Pi-S-NI), I (Pi-R/Pi-S) (i.e., Pi-R-I relative to Pi-S-I), and NI (Pi-R/Pi-S) (i.e., Pi-R-NI relative to Pi-S-NI). All values obtained in the qRT-PCR analysis were expressed as the mean ± SD (standard deviation of the mean), and based on 5 independent experiments (i.e., 5 biological repeats), independent samples *t*-test (*n* = 5) was applied in the SPSS Statistics 17.0 context to assess the significance of differences between the means (*p < 0.05 and **p < 0.01).

### Construction of *mfs1*-knockout mutant (Δ*mfs1*) from Pi-R

The mutant Δ*mfs1* was derived from its parental strain Pi-R by introduction of *mfs1*- knockout cassette into the DMI-resistant *P. italicum* protoplasts that were prepared according to the method of Zhao et al. [104]. Double-joint PCR was performed to construct the desirable knockout cassette, also according to the method of Zhao et al. [104]. In detail, the hygromycin phosphotransferase (*hph*) resistance gene (approximately 2.1 kb) from the plasmid pTFCM (generously provided by Dr. Daohong Jiang, Huazhong Agricultural University, China) was inserted between the upstream and downstream flanking sequences of *mfs1* gene (approximately 2.0 kb in total) from the Pi-R genomic DNA. The knockout cassette was introduced into wild-type Pi-R protoplasts as described by Zhao et al. [104], and thus the *Pimfs1* gene was replaced by the *hph* resistance gene.

## Supplementary information

**Additional file 1: Figure S1.** PDA-based EC_50_ determination against prochloraz for two *Penicillium italicum* strains (Pi-R and Pi-S) with different response to the DMI prochloraz.

**Additional file 2: Table S1.** EC_50_ values of Pi-R and Pi-S against the fungicides belonging to DMI, benzimidazole and phenylpyrrole classes.

**Additional file 3: Table S2.** Primers used in the present qRT-PCR validation.

**Additional file 4: Figure S2.** Genome mapping analysis for the clean reads from the present 4 RNA-seq libraries, i.e., Pi-R-I (a), Pi-R-NI (b), Pi-S-I (c), and Pi-S-NI (d).

**Additional file 5: Table S3.** Summary of DEGs in the comparison between prochloraz-induced and no-induced Pi-R strains.

**Additional file 6: Table S4.** Summary of DEGs in the comparison between prochloraz-induced and no-induced Pi-S strains.

**Additional file 7: Table S5.** Summary of DEGs in the comparison between Pi-R and Pi-S strains under prochloraz-induced conditions.

**Additional file 8: Table S6.** Summary of DEGs in the comparison between Pi-R and Pi-S strains without prochloraz induction.

**Additional file 9: Table S7.** Summary of distribution (hit records) of 19 selected DEGs towards GO enrichments in the present 4 comparisons.

**Additional file 10: Table S8.** FPKM values of the additional 4 prochloraz-responsive unigenes in the present comparisons.

**Additional file 11: Figure S3.** *GAPD*-based qPCR validation of 19 prochloraz-responsive DEGs including drug transporter genes (a), ergosterol biosynthesis-related genes (b), MAPK signaling pathway genes (c), and Ca^2+^ signal transduction genes (d).

**Additional file 12: Figure S4.** Comparison of prochloraz EC_50_ values between Pi-R (wild type) and its *mfs1*-knockout mutant (Δ*mfs1*).

**Additional file 13: Table S9.** Comparative analysis of prochloraz-responsive gene expression profiles between the *P. italicum* and *P. digitatum* transcriptomes.

## Data Availability

RNA-seq data discussed in this article are publicly available at the NCBI database under accession number PRJNA421419.

## Abbreviations

ABC: ATP-binding cassette
BP: Biological process
CaM: Ca^2+^/calmodulin
CaMK: Ca^2+^/calmodulin-dependent kinase
CC: Cellular component
CWI: Cell wall integrity
DEG: Differentially expressed gene
DMI: Demethylation inhibitor
ERG: Ergosterol
FPKM: Fragments Per Kilobase per Million mapped reads
GAPD: Glyceraldehyde-3-phosphate dehydrogenase
GO: Gene Ontology
KEGG: Kyoto Encyclopedia of Genes and Genomes
MAP: Mitogen-activated protein
MAPK: Mitogen-activated protein kinase
MAPKK: Mitogen-activated protein kinase (MAPK) kinase
MAPKKK: Mitogen-activated protein kinase (MAPK) kinase kinase
MATE: Multidrug and toxic compound extrusion
MF: Molecular function
MFS: Major facilitator superfamily
PCR: Polymerase chain reaction
PDA: Potato dextrose agar
PDB: Potato dextrose broth
qPCR: Quantitative real-time PCR
RNA-seq: RNA sequencing
