# Supplementary material for: Transcriptome analysis of fungicide-responsive gene expression profiles in two Penicillium italicum strains with different response to the sterol demethylation inhibitor (DMI) fungicide prochloraz
Source: BMC Genomics. 2020 Feb 12;21:156. doi: 10.1186/s12864-020-6564-6 (PMC7017498; doi:10.1186/s12864-020-6564-6)
Supplement: Supplementary file 1 — Additional file 1: Figure S1. PDA-based EC50 determination against prochloraz for two Penicillium italicum strains (Pi-R and Pi-S) with different response to the DMI prochloraz. [file 12864_2020_6564_MOESM1_ESM.doc]

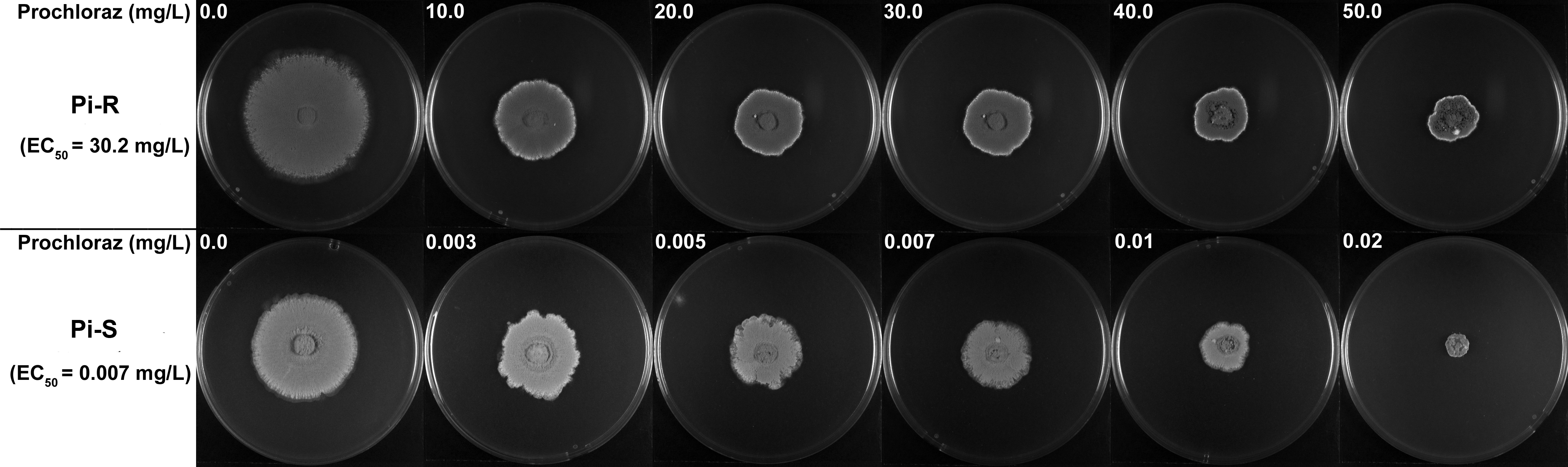


**Additional file 1: Figure S1.** PDA-based EC_50_ determination against prochloraz for two *Penicillium italicum* strains (Pi-R and Pi-S) with different response to the DMI fungicide prochloraz.
