# Supplementary material for: Transcriptome analysis of fungicide-responsive gene expression profiles in two Penicillium italicum strains with different response to the sterol demethylation inhibitor (DMI) fungicide prochloraz
Source: BMC Genomics. 2020 Feb 12;21:156. doi: 10.1186/s12864-020-6564-6 (PMC7017498; doi:10.1186/s12864-020-6564-6)
Supplement: Supplementary file 4 — Additional file 4: Figure S2. Genome mapping analysis for the clean reads from the present 4 RNA-seq libraries, i.e., Pi-R-I (a), Pi-R-NI (b), Pi-S-I (c), and Pi-S-NI (d). [file 12864_2020_6564_MOESM4_ESM.doc]

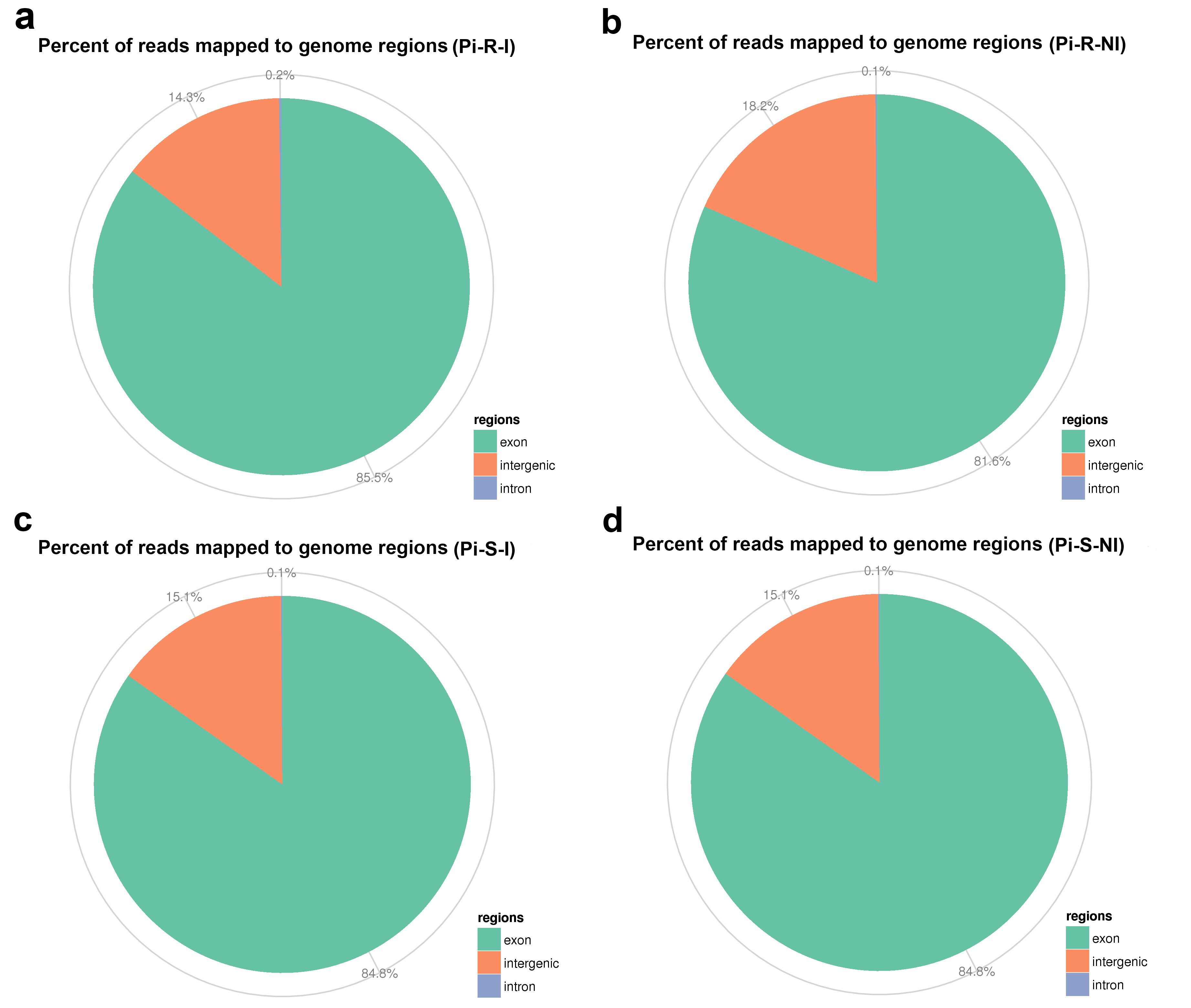


**Additional file 4: Figure S2.** Genome mapping analysis for the clean reads from the present 4 RNA-seq libraries, i.e., Pi-R-I (a), Pi-R-NI (b), Pi-S-I (c), and Pi-S-NI (d).
