# Supplementary material for: Transcriptome analysis of fungicide-responsive gene expression profiles in two Penicillium italicum strains with different response to the sterol demethylation inhibitor (DMI) fungicide prochloraz
Source: BMC Genomics. 2020 Feb 12;21:156. doi: 10.1186/s12864-020-6564-6 (PMC7017498; doi:10.1186/s12864-020-6564-6)
Supplement: Supplementary file 11 — Additional file 11: Figure S3. GAPD-based qPCR validation of 19 prochloraz-responsive DEGs including drug transporter genes (a), ergosterol biosynthesis-related genes (b), MAPK signaling pathway genes (c), and Ca2+ signal transduction genes (d). [file 12864_2020_6564_MOESM11_ESM.doc]

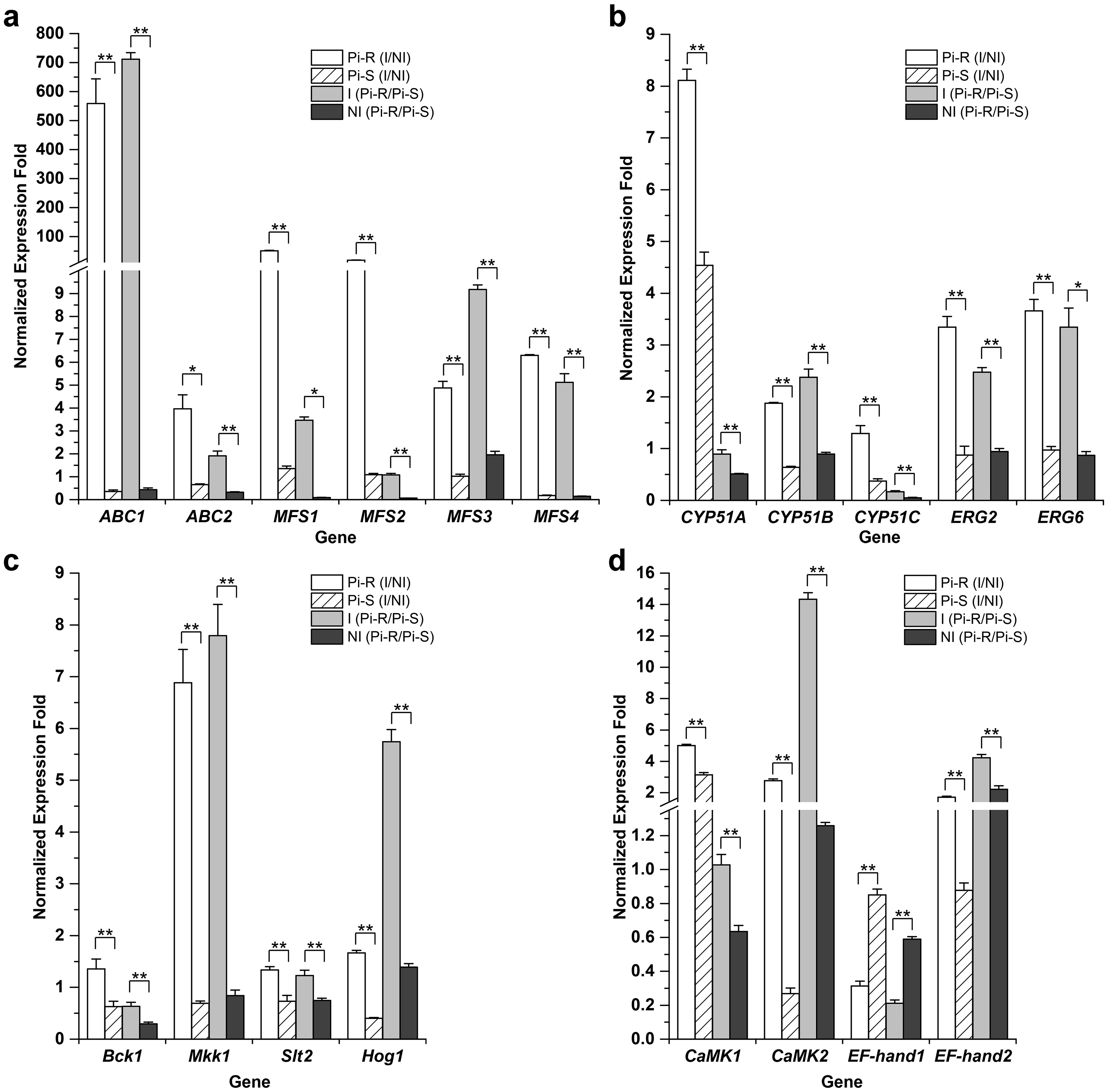


**Additional file 11: Figure S3.** *GAPD*-based qPCR validation of 19 prochloraz-responsive DEGs including drug transporter genes (a), ergosterol biosynthesis-related genes (b), MAPK signaling pathway genes (c), and Ca^2+^ signal transduction genes (d).
