# Supplementary material for: Transcriptome analysis of fungicide-responsive gene expression profiles in two Penicillium italicum strains with different response to the sterol demethylation inhibitor (DMI) fungicide prochloraz
Source: BMC Genomics. 2020 Feb 12;21:156. doi: 10.1186/s12864-020-6564-6 (PMC7017498; doi:10.1186/s12864-020-6564-6)
Supplement: Supplementary file 12 — Additional file 12: Figure S4. Comparison of prochloraz EC50 values between Pi-R (wild type) and its mfs1-knockout mutant (Δmfs1). [file 12864_2020_6564_MOESM12_ESM.doc]

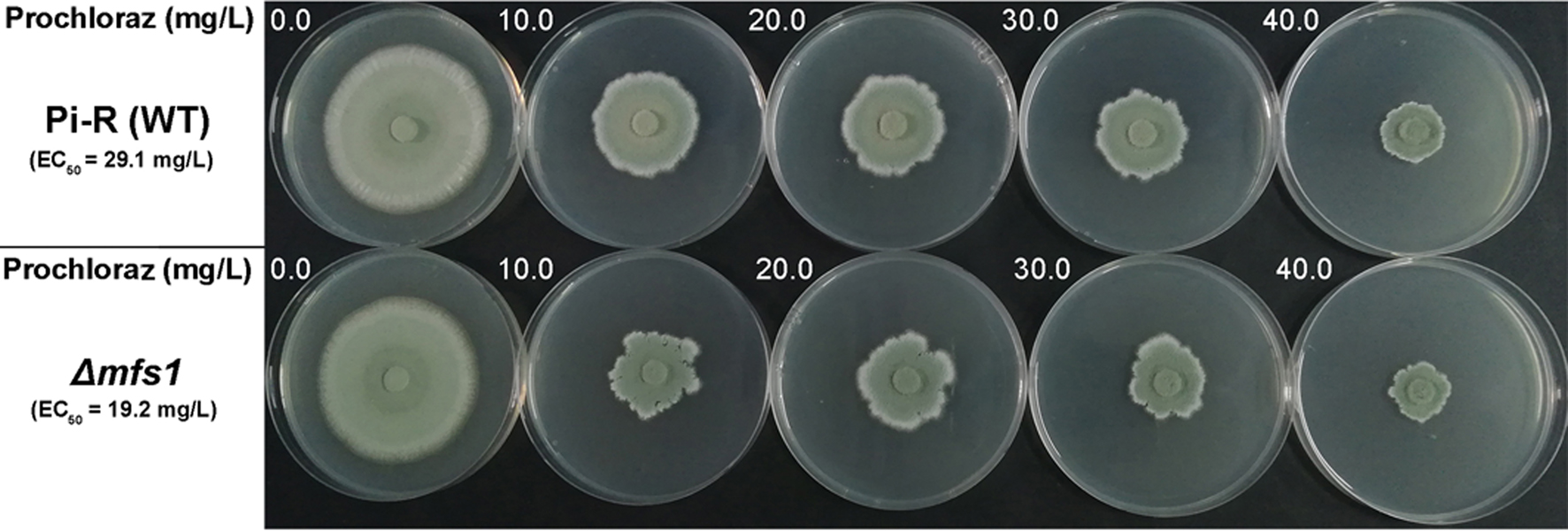


**Additional file 12: Figure S4.** Comparison of prochloraz EC_50_ values between Pi-R (wild type) and its *mfs1*-knockout mutant (Δ*mfs1*).
